# Potential Enhancement of Metformin Hydrochloride in Lipid Vesicles Targeting Therapeutic Efficacy in Diabetic Treatment

**DOI:** 10.3390/ijms22062852

**Published:** 2021-03-11

**Authors:** Emmanuel Chekwube Ossai, Augustine Chidi Madueke, Benjamin Emenike Amadi, Martins Obinna Ogugofor, Audu Mumuni Momoh, Charles Odilichukwu R. Okpala, Chioma Assumpta Anosike, Obioma Uzoma Njoku

**Affiliations:** 1 Department of Biochemistry, Faculty of Biological Sciences, University of Nigeria, Nsukka 410001, Enugu, Nigeria; chidi.madueke@unn.edu.ng (A.C.M.); emenike.amadi.pg.78178@unn.edu.ng (B.E.A.); obinna.ogugofor.pg76810@unn.edu.ng (M.O.O.); chioma.anosike@unn.edu.ng (C.A.A.); obioma.njoku@unn.edu.ng (O.U.N.); 2 Department of Pharmaceutics, Faculty of Pharmaceutical Sciences, University of Nigeria, Nsukka 410001, Enugu, Nigeria; audu.momoh@unn.edu.ng; 3 Faculty of Biotechnology and Food Science, Wrocław University of Environmental and Life Sciences, 51-630 Wrocław, Poland

**Keywords:** drug development, bioavailability, therapeutic potentials, diabetes, biotechnology

## Abstract

The potential enhancement of metformin hydrochloride (MH) loaded in lipid vesicles targeting therapeutic efficacy on alloxan-induced diabetic rats was investigated. This involved lipid vesicles formulated with homogenously distributed nano-sized particles by a novel integrated process of multiple emulsification by membrane and solvent evaporation. The average diameter of the water-in-oil (W_1_/O), W_1_/O/W_2_ emulsion droplets, and lipid vesicles was 192 nm, 52 µm, and 173 nm, respectively. The entrapment yield of metformin hydrochloride (MH) in the prepared lipid vesicles was 40.12%. The metformin hydrochloride-loaded lipid vesicles (MH-LLVs) sustained the release of the entrapped drug over a 12-h period and reduced the plasma glucose level of diabetic rats by 77.4% compared with free MH solution (2-h period and 58.2%, respectively) after one week post-diabetic treatment through oral administration of MH-LLV and the free drug. The remarkable improvement in the biochemical parameters recorded in the MH-LLV-treated animals compared with those that received free MH solutions depicted an enhanced kidney function, liver function, as well as oxidative stress status. Pancreatic histology depicted a pancreas with intralobular ducts (ID) and exocrine secretory acini that characterize an intact pancreas, which suggests the ability of the MH-LLVs to restore pancreatic cells to normal, on a continued treatment. Overall, MH-LLV appears an encouraging extended-release formulation with enhanced bioavailability, sustained release, and improved antihyperglycemic potentials.

## 1. Introduction

Metformin hydrochloride (MH), in addition to its Food and Drug Administration (FDA) approval in the United States, is among the biguanide first-line drugs of choice for diabetes management [1,2]. Noting its extremely safe profile, it became of use as Glucophage^®^ by 1995 and among oral antidiabetics in the model list of essential medicines by the World Health Organization (WHO) of the United Nations by 2010 [2,3]. MH delivers key functions such as facilitating the decrease in hepatic glucose output as well as supporting/upregulating the insulin-engineered glucose uptake by cells. Additionally, it can diminish the absorption of glucose in the gut and downregulate the plasma concentration of free fatty acids, thereby cutting down the amount of substrate available for glucose production from non-carbohydrate sources [4]. Metformin has some drawbacks, such as the induction of lactic acidosis/hepatotoxicity, which is clinically possible in less than 1% of patients. It can also perform as an insulin-sensitizing agent. This function might be through the activation of adenosine monophosphate-dependent kinase, obtainable in the liver/muscle tissues [3]. Moreover, without inducing overt hypoglycemia, MH can reduce the plasma glucose concentration. The bioavailability of MH appears relatively low at about 50–60% [5] after oral administration, with urinary excretion of 30% to 50% of the unchanged form of the drug, while about 30% is removed in feces within 24 h [6].

Particularly, the conventional method of treatment of diseases requires a high level of medication, which has the propensity to generate a high level of systemic toxicities. Moreover, to maintain low serum drug levels, there would be concerns of under-dosing, which could result in drug resistance [7]. Prevention of such adverse effects could be achieved by a localized delivery system of medication. The oral route of drug delivery has always been associated with, for instance, the challenge of rapid dissolution in the gastrointestinal tract, which brings about the inadequacy of blood level peaks. Despite the concerns associated with the blood level, when the concentration begins to decrease, it would be necessary for there to be an intake of an additional drug dose, particularly within a few hours, to maintain the optimum blood level. Such constant drug dosing remains problematic owing to the irritable properties of some drugs within the stomach and the associated incidence of adverse effects. To combat the above challenges, given the need for sustained release of drugs, enormous research interests have been spurred. Chief among them is the application of nanotechnology in sustained and targeted delivery of pharmaceutics.

As an oral antihyperglycemic agent, MH suffers from poor bioavailability due to slow or incomplete absorption. Consequently, repeated administration of high doses is required to establish treatment. Reduction in patience compliance and occurrence of side effects have been reported as some of the problems associated with the use of repeated high doses of MH [8,9]. More importantly, lactic acid accumulation, a fatal metabolic condition, has been reported with biguanides at extreme overdose [8]. Designing and developing a novel formulation strategy with a sustained release property for MH is desirable for improving drug bioavailability and reducing frequent dosing, gastrointestinal tract (GIT) effects, and secondary toxicities [5,10], which would enhance the general therapeutic potentials of the drug. Formulating an extended-release system of MH has been approached via microencapsulation methods, namely: a) emulsion solvent evaporation employing low- and high-permeability methacrylic resins (eudragit), cellulose, and pectin; and b) ionic gelation employing alginate/gum karaya and cordia gum/gellanum gum [5,11,12,13]. Furthermore, controlled-/extended-release formulations of MH have been developed for once-daily dosage, having similar pharmacokinetic profiles to immediate-release formulations [14,15,16,17] but with improved patient compliance. Similarly, clinical studies have been conducted with a delayed-release formulations of MH [18,19]. However, the reported methods are ineffective due to the large size (micro-scale) of the formulated carriers, which could still hamper or impede optimum absorption in the GIT. Few hundred nanometer-sized carriers of MH have been reported using niosomes [20]. However, there is still a need for smaller-sized carriers for improved bioavailability and ease of extravasation into the tissues.

Herein, the potential enhancement of metformin hydrochloride loaded in lipid vesicles (MH-LLVs) targeting therapeutic efficacy on alloxan-induced diabetic rats was investigated. The lipid vesicles encapsulating MH in high yield, with homogenously distributed nano-sized particles by an integrated process of multiple emulsification and by membrane and solvent evaporation, employed a low concentration of Tween^®^ 80 as the continuous phase emulsifier. Specifically, in vitro drug release and in vivo effects of the lipid vesicle-encapsulated MH were investigated on the diabetic rat model.

## 2. Results

### 2.1. Preparation and Characterization of Metformin Hydrochloride-Loaded Lipid Vesicles

**Figure 1:**
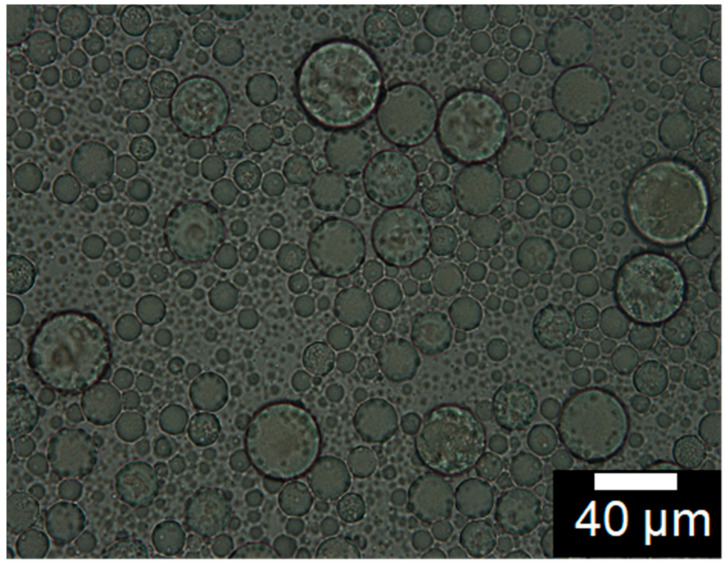
Photomicrograph of W_1_/O/W_2_ multiple emulsion.

**Figure 2:**
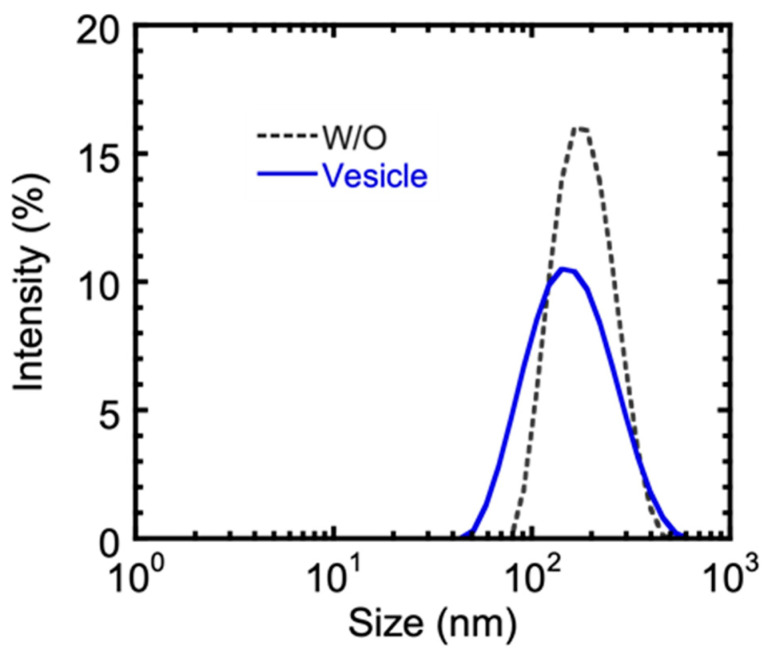
Intensity size distribution of primary water-in-oil emulsion and the resulting lipid vesicle.

**Figure 3:**
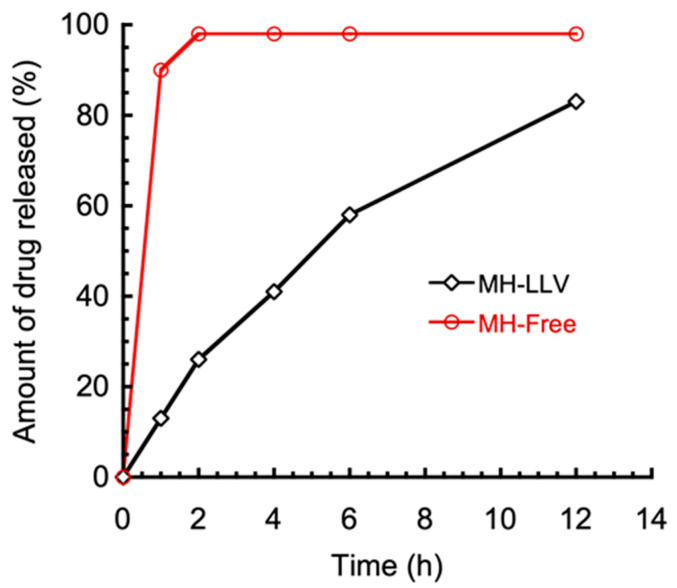
The in vitro drug release study of metformin hydrochloride-loaded lipid vesicles (MH-LLVs).

Lipid vesicles entrapping metformin hydrochloride in high yield were prepared by multiple emulsification method, coupled with solvent evaporation. The W_1_/O (primary) emulsions were formed by treating the internal water phase together with the continuous oil phase with ultrasonic waves. A photomicrograph of the W_1_/O/W_2_ multiple emulsions is shown in Figure 1. The primary emulsion formed was used as a dispersed phase in the secondary membrane emulsification, which formed the multiple W_1_/O/W_2_ emulsion droplets. The average size of the multiple emulsion droplets was about 52 mm, with the sizes distributed between 35 and 72 mm. The intensity size distribution of the primary water-in-oil emulsion and the resulting lipid vesicle is shown in Figure 2. The peak diameter of the initial water droplets of the primary emulsion (192 nm) appears similar to that of the resulting vesicles (173 nm), with an entrapment yield of metformin in the lipid vesicles of 40.12 ± 1.44%. The in vitro drug release study of metformin hydrochloride-loaded lipid vesicles is shown in Figure 3. The drug-loaded vesicles can achieve a sustained drug release over a 12-h period, with an increasing amount of drug release during the first 6 h. A maximum drug release of 83%, intriguingly, appears to have been achieved after 12 h.

### 2.2. Biochemical Studies

#### 2.2.1. The Effects of Metformin Hydrochloride-Loaded Lipid Vesicles on the Body Weight and Serum Glucose Level of Alloxan-Induced Diabetic Rats

**Figure 4:**
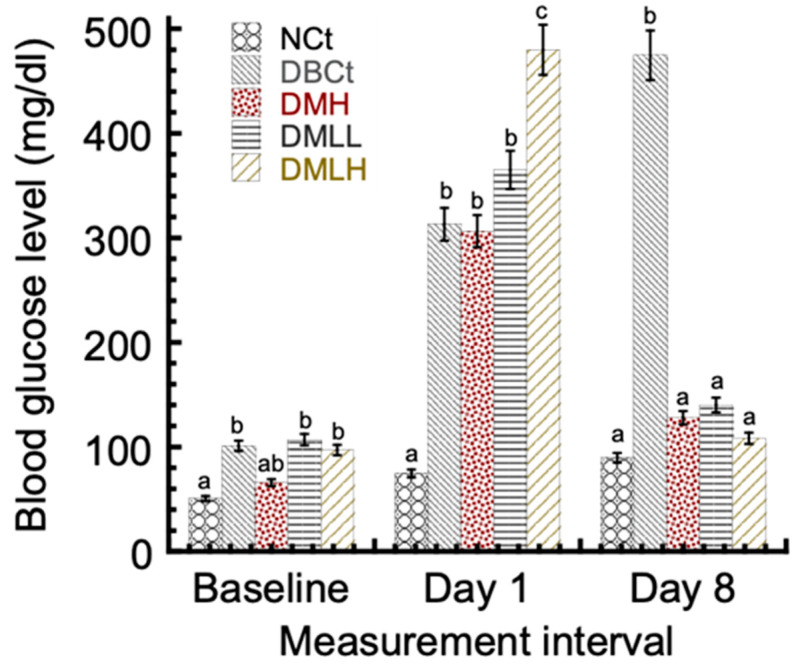
Effect of metformin hydrochloride-loaded lipid vesicles on the blood glucose level of rats. *NCt: Normal control; DBCt: Diabetic control; DMH: Diabetic + 5 mg/kg body weight free MH; DMLL: Diabetic + low dose (2.5 mg/kg body weight MH-LLV); DMLH: Diabetic + high dose (5 mg/kg body weight MH-LLV). Data are shown as mean ± SD of five animals. “a–c”: Presence of the same letters in a column indicates no significant difference (*p* > 0.05), while different superscripts in a column show significant difference (*p* < 0.05).

The body weights of the animals in the NCt-, MH-, and MH-LLV-treated groups were found to improve significantly compared with a diabetic control (data not shown). The blood glucose levels of the animals were estimated using a glucometer. Between days 1 and 8, the blood glucose levels of the diabetic control, DBCt, were noticeably (*p* < 0.05) higher than the normal control, NCt, and diabetic-treated groups (Figure 4). The blood glucose levels of the NCt did not significantly differ (*p* > 0.05) from the DMH, DMLL, and DMLH groups. However, the reduction in blood glucose levels seems more pronounced in the groups treated with lipid vesicle-entrapped drug compared with the free drug-treated group DMH. In particular, the DMLH group obtained the highest blood glucose reduction of 77.4% compared with the 58.2% blood glucose reduction seen in the DMH group.

#### 2.2.2. The Effect of Metformin Hydrochloride-Loaded Lipid Vesicles on the Serum Lipid Profile and Liver Enzymes of Alloxan-Induced Diabetic Rats

The effect of metformin hydrochloride-loaded lipid vesicles (MH-LLVs) on the serum lipid profile and liver enzyme status of the experimental animals is shown in Table 1. A significant difference (*p* < 0.05) in total cholesterol can be seen in the DBCt group, somewhat higher when compared with the NCt, and in the treated groups. Similarly, the level of total cholesterol appears significantly (*p* < 0.05) higher in the free drug-treated group (DMH) compared with the vesicle-entrapped-drug-treated groups. However, the total cholesterol levels in the animals treated with a low dose (DMLL) resembled (*p* > 0.05) those of rats treated with a high (DMLH) dose of the carrier-entrapped drug. The level of triacylglycerols (TAGs) was also significantly (*p* < 0.05) higher in the DBCt group compared with those of the NCt and the drug-treated groups. However, the TAG levels were similar (*p* > 0.05) across the drug-treated groups, even though the DMH group obtained a higher level than DMLL and DMLH. Conversely, the level of high-density lipoprotein (HDL)-cholesterol in the DBCt group was significantly (*p* < 0.05) lower compared with those of the NCt and drug-treated groups. For HDL-cholesterol, the level in the DMH group resembled (*p* > 0.05) those in the carrier-entrapped-drug-treated groups, even though DMLH obtained the highest level of HDL-cholesterol compared with the others. Nonetheless, low-density lipoprotein (LDL)-cholesterol reached considerably (*p* < 0.05) higher values in the DBCt group compared with the NCt group and the drug-treated groups, even though the values appeared lowest in the vesicle-entrapped-drug-treated groups than in the DMH group. With respect to the liver function tests, aspartate aminotransferase (AST), alanine aminotransferase (ALT), and alkaline phosphatase (ALP) obtained significantly higher (*p* < 0.05) activities in the DBCt group compared with the NCt group and the drug-treated groups. AST and ALT operated at significantly (*p* < 0.05) lower levels in the DMLH group compared with the DMH group. However, the ALP levels in the DMH group resembled (*p* < 0.05) those of the DMLH group. The total bilirubin levels in the DBCt group were significantly (*p* < 0.05) higher compared with those of the NCt group and the drug-treated groups. The level, however, was lowest in the DMLL group compared with the DMH and DMLH groups.

#### 2.2.3. The Effect of Metformin Hydrochloride-Loaded Lipid Vesicles on the Levels of Oxidative Stress in Alloxan-Induced Diabetic Rats

The effect of metformin hydrochloride-loaded lipid vesicles (MH-LLVs) on the oxidative stress status of alloxan-induced diabetic rats is shown in Table 2. Glutathione (GSH) was at significantly (*p* < 0.05) higher levels in the DMLH group compared with the DMH and DBCt groups. The activities of superoxide dismutase (SOD) and catalase (CAT) in the DMLH group resembled (*p* > 0.5) those in the DMH group. However, their activities were lowest, to a significant degree (*p* < 0.05), in the DBCt group compared with other groups. The activities of glutathione peroxidase (GPx) in the DBCt group operated at significantly (*p* < 0.05) lower levels compared with those of the NCt group and the drug-treated groups. Similarly, the GPx activity in the DMLH group was significantly higher (*p* < 0.05) compared with that in the DMH group. The levels of lipid peroxidation [malondialdehyde (MDA)] were highest in the DBCt and DMH groups compared with the NCt group and the vesicle-entrapped-drug-treated groups. For the vesicle-entrapped-drug-treated groups, MDA was at a significantly (*p* < 0.05) lower level compared with the DMH group.

#### 2.2.4. The Effect of Metformin Hydrochloride-Loaded Lipid Vesicles on the Kidney Profile and Serum Electrolyte Levels in Alloxan-Induced Diabetic Rats

Table 3 shows the renal function parameters and the serum electrolyte levels in the experimental animals. Creatinine decreased at a significant level (*p* < 0.05) in the DBCt group relative to the NCt group and the drug-treated groups. There were significant increases (*p* < 0.05) in the creatinine levels in the DMH, DMLL, and DMLH groups relative to the DBCt group. The creatinine in the DMH group operated at significantly (*p* < 0.05) lower levels relative to the lipid vesicle-entrapped-drug-treated groups, with DMLH having the peak creatinine values. Urea levels were significantly higher (*p* < 0.05) in the DBCt group relative to animals in the NCt group. However, the urea levels of animals in the metformin-treated groups were lower than those in the DBCt group, with DMLH-treated animals having significantly lower levels of urea than the DBCt group.

The sodium ions in the DBCt animal group operated at significantly (*p* < 0.05) lower levels than in the NCt group, and also in the metformin-treated groups. However, the difference in sodium ion levels of vesicle-entrapped-drug-treated groups resembled (*p* > 0.05) those of the free drug-treated group. Furthermore, the difference in sodium ion levels between the NCt group also resembled (*p* > 0.05) those of metformin-treated groups. The levels of potassium, chloride, and bicarbonate ions in the DBCt animal group were lower, to a significant degree (*p* < 0.05), than those in the NCt group and the metformin-treated groups. Additionally, the difference in the potassium, chloride, and bicarbonate ions levels between the NCt group and the groups treated with metformin were also similar (*p* > 0.05).

**Figure 5:**
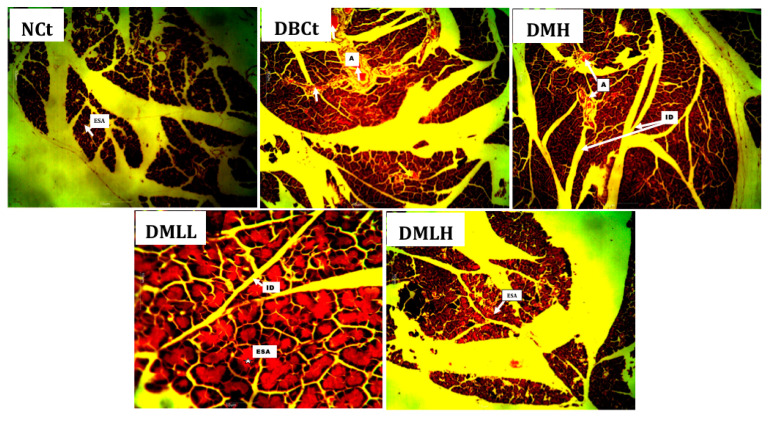
Photomicrograph of the histopathological evaluation of the experimental animals’ pancreatic tissues after the experimental period elapsed.

Histological investigation of pancreatic tissues showed that NCt animals had intact exocrine secretory acini (ESA) (Figure 5). The DBCt group animals showed a pancreas with severe angiectasis. The DMH group showed a histological section of the pancreas with severe angiectasis, as observed in DBCt, which are inconsistent dilated spaces that contain red blood cells and plasma with a vanishingly small endothelial cell lining. The pancreatic tissues of the DMLL and DMLH groups showed intact pancreases with clear morphological representation: intralobular ducts and exocrine secretory acini.

## 3. Discussion

Lipid vesicles are drug delivery carrier systems that offer promising benefits in pharmacology, especially in terms of reducing the dosing frequency of drugs, increasing their bioavailability, preventing the degradation of drug molecules, specifically by the unpleasantly rough gastric umbworld, improving receptor targeting, and also reducing the associated unwanted effects of pharmacological agents [21]. Despite the progress recorded in improving on the adverse effects and low bioavailability of metformin as a first-choice oral antidiabetic drug, there are still challenges of formulating a few hundred nanometer-scaled carriers with high entrapment yield of the bioactive molecule. The evaluation of metformin hydrochloride-loaded lipid vesicles for antidiabetic activity in alloxan-mediated diabetic animals demonstrated a considerable lowering in hyperglycemia, sustained release of the encapsulated drug, and improvement in its bioavailability compared with free metformin hydrochloride. This underscores the reason why metformin hydrochloride-loaded lipid vesicles (MH-LLVs) were prepared using the multiple emulsion and solvent evaporation integrated method. In particular, the multiple emulsions were prepared by membrane emulsification [22].

In the current work, the size distribution of the W_1_/O/W_2_ multiple emulsion droplets (refer to Figure 1) ranged from 35 to 72 mm with an average size of 52 mm. This was comparable to multiple emulsion droplet sizes in our previous report [23], specifically for the entrapment of calcein in lipid vesicles. The size of the lipid vesicles is of great importance in terms of their application as carrier vehicles for drug delivery [24]. Herein, the modal size of the lipid vesicles resulting after the removal of organic solvent from the W_1_/O/W_2_ multiple emulsion droplets was 173 nm and was comparable to the modal size of the initial water droplets (192 nm) of the primary emulsion (refer to Figure 2). This would suggest that the removal of organic solvent in the oil phase of W_1_/O/W_2_ emulsion droplets leads to the formation of lipid vesicles via self-assembling of the bilayer-forming lipids around each water droplet of the primary W_1_/O emulsion. Lipid vesicles with sizes in the submicron dimension were reported to achieve faster transfer from blood vessels to target sites [25].

To achieve a desired therapeutic level of drugs, efficient entrapment is needed. However, an adequate amount of lipids is required to obtain such efficient entrapment. Efficient entrapment has, therefore, become a central challenge and priority for good lipid vesicle engineering. The entrapment yield of 40.12% for MH in the prepared lipid vesicles of the current work appeared higher than that reported elsewhere [5] specific to polymeric particles formed via the nanoprecipitation method. Emulsifiers whose molecular weights are high tend to produce more considerable entrapment of hydrophilic bioactive(s), which might corroborate their ability to minimize efflux of the entrapped molecules [22]. According to Kuroiwa et al. [22], the use of 3.0 wt % (*w*/*v*) Tween^®^ 80 with a relative molecular weight of 1310 as an emulsifier in the external water phase resulted in an entrapment yield of as low as 20% for calcein. Previously, Ossai et al. [23] suggested that the entrapment yield of calcein increased with a decrease in Tween 80 concentration, which conforms with our present study in which an appreciable entrapment of 40.12% for MH was obtained at low concentration (0.1 wt %) of Tween^®^ 80 as the external water phase emulsifier. The lower entrapment yields of the current work’s formulation compared with that previously established by Ossai et al. [23], specific to the entrapment of calcein, could be due to either efflux or leakage of the entrapped material from the internal aqueous phase to the continuous aqueous phase, which might have partially taken place as the solvent evaporation proceeded. Specifically, the lower relative molecular weight of MH (165.6 g/mol) might be an explanation for this occurrence (of efflux or leakage of the entrapped material), compared with that of calcein (622.6 g/mol), of the current work. In addition, besides the use of polymer-based surfactants such as proteins as shown by Garti [26], there is the need to curb the leakage of entrapped materials between the inner and outer aqueous phases. The use of protein emulsifiers, however, would limit the wider applications of carrier products, which would be due to their potentials of eliciting allergic reactions in some individuals.

The in vitro release of MH (refer to Figure 3) showed a sustained release of the drug by the lipid vesicles over 12 h compared with the release of the free drug solution, which lasted for about 2 h. The drug release process followed two phases: the first phase was characterized by a high release rate known as the burst effect. The second phase recorded a slower rate of drug release from the vesicles as time elapsed. The sustained-release property of the formulation could follow which demonstrates the ability of the formulation to retain the drug for a prolonged time. Furthermore, the lipid vesicles entrapping metformin HCl were found to be stable for 28 days at 4 °C (data not shown).

Diabetic condition is associated with loss of body weight due to breakdown of proteins in peripheral tissues [27]. In our study, the body weights of rats in the diabetic control groups were found to decrease significantly compared with the NCt and drug-treated groups. The antidiabetic results of the current work indicate that the metformin hydrochloride-loaded lipid vesicle significantly (*p* < 0.05) reduced hyperglycemia in the alloxan-induced diabetic rats in a sustained pattern compared with the bioactive compound alone (free metformin hydrochloride). This could be attributed to the improved bioavailability of the drug-mediated entrapment into the lipid vesicles. Lipid vesicles can increase the bioavailability of drug molecules via integration of the lipid component with the cell membrane, or following the transappendageal routes [28]. The ability of lipid vesicles to make use of the paracellular and transappendageal pathways would associate with the excipients used during the formulation [21]. Comparing the standard control (DMH) at 5 mg/kg body weight, the metformin hydrochloride-loaded lipid vesicles achieved serum glucose lowering by 77.4%, whereas the standard drug (metformin hydrochloride) reduced the serum glucose level by 58.2%. This suggests that the lipid vesicle formulation of metformin hydrochloride might have an improved antidiabetic potential over the free metformin hydrochloride (refer to Figure 4).

The total serum lipids (total cholesterol, triacylglycerol, and low-density lipoprotein cholesterol) levels in treated animals were lower than in the diabetic control (DBCt) group, but high-density lipoprotein concentrations of the treated groups were significantly higher relative to the DBCt group. Animals that received MH-LLVs at the dosage of 5 mg/kg body weight (DMLH group) recorded elevated HDL levels and lower LDL, TAG, and total cholesterol (T.CHOL) levels relative to the standard control (DMH), showing that the administration of MH-LLVs produces hypoglycemic and hypolipidemic effects and may prevent cardiovascular abnormalities (refer to Table 1). An increase in the risk factor of cardiovascular disease correlates with an increase in serum total cholesterol, LDL, TAG level, and atherogenic index level and a decrease in HDL concentrations. A similar decrease in serum total lipids was also reported in the literature [29] and correlates with a decrease in circulating free fatty acids [30]. Such a decrease is expected in the case of accumulation of fat in adipocytes, both by synthesis and decreased release of fatty acids.

Observed activities of liver enzymes were elevated in the diabetic controls relative to the normal control. These high levels of liver enzyme activities indicate possible leakage or discharge from hepatocytes and, thus, an apparent loss of biological integrity of liver cell membranes [31]. Administration of MH-LLVs to diabetic groups mildly adjusted the elevated activities of these enzymes toward normal levels, especially at the highest dose of metformin-containing vesicles (refer to Table 1). The adjustment, therefore, suggests that on continued treatment, MH-LLVs may possibly stabilize the cell membrane as well as restore hepatic tissue health, as documented in the literature [32]. Compromise of liver health in the diabetic control was also established by high levels of bilirubin in the normal control group animals and agrees with the report in the literature [33]. The increased level of bilirubin may be due to the failure of the mechanism involved in conjugation or uptake by the liver. However, MH-LLV administration slightly lowered the total bilirubin level after administration for 7 days slightly more than free MH solution, indicating an improvement in liver health [33].

Diabetes is basically associated with an increase in free radical formation and diminished antioxidant potential [34]. The increase in GSH levels coupled with the increase in GPx, CAT, and SOD activities in serum and the decrease in MDA levels in tissues of animals in the normal control group and the drug-treated groups demonstrate improvement in oxidative stress and, by extension, the diabetic condition upon treatment with the drug. Moreover, MH-LLV-treated animals had better antioxidative indices compared with the animals treated with the free form of the drug (refer to Table 2). The antioxidant effect of MH-LLVs could be explained by two mechanisms. It could be proposed that MH-LLVs could act by preventing protein glycosylation and peroxidation by interacting with free radicals and minimizing their damaging effects. Secondly, MH-LLVs may possibly initiate the synthesis of antioxidant enzymes. Moreover, previous studies have reported the inhibition of glycogenolysis and gluconeogenesis by MH through the inhibition of protein kinases and the three primary enzymes of gluconeogenesis (PEP carboxykinase and glucose-6-phosphatase). MH was also reported to induce enzyme expression of antioxidant enzymes at the transcriptional level [35]. Furthermore, GSH plays a pivotal role in regulating the body’s antioxidant defense mechanisms. Unfavorable alterations in the GSH level of the living system pose some unwanted metabolic outcomes [36]. Reduction in the GSH level in the diabetic rats and its later return to near normal in MH-LLV-treated groups underscore the antioxidant potentials of the formulation. It is suggested that MH-LLV mediates its antioxidant effect through increases in the level of GSH and elimination of free radicals via increases in the activities of antioxidant enzymes [37].

High levels of urea in the diabetic animals indicate possible renal dysfunction. Within the short treatment period, metformin treatment could reduce serum urea levels in animals, suggestive of amelioration of renal damages due to diabetes. High urea concentration in diabetic animals was predicted to be due to upregulation of gluconeogenesis as an alternative glucose source due to insulin deficiency [38]. Gluconeogenesis is maintained by proteolysis and the process-mediated release of glucogenic amino acids, which are deaminated in the liver, producing urea [38]. Creatinine levels in the sera of diabetic non-treated animals were lower than those in the MH-LLV-treated groups, which lends credence to the findings that lower creatinine in serum is associated with insulin resistance [39]. Thus, it is reasonable to suggest that the ability of the lipid vesicle-entrapped metformin to reduce insulin resistance effectively could be reasonable, in part, associated with the increase in creatinine levels observed in the MH-LLV-treated diabetic animals relative to the diabetic free MH-treated group and the diabetic non-treated ones (refer to Table 3).

The high volume of metabolites and their increased excretion through the kidneys above the normal homeostatic range lead to an imbalance in the electrolyte concentration [40]. It is logical to think that high losses of electrolytes and water, as seen in diabetics, may cause a reduction in serum electrolytes and, thus, result in the excretion of electrolytes by parietal cells [41], which can explain the observed reduction in the serum sodium and potassium ions of the diabetic control relative to the normal control (refer to Table 3). The increase in the electrolyte concentrations after the administration of free MH and MH-LLV indicates that the formulation could effectively restore the altered extracellular fluid electrolyte level of the diabetic treated animals [41]. More so, the increase in serum chloride and bicarbonate ions levels in MH-LLV-treated animals relative to diabetic control animals as seen in this study suggests that the formulation enhances rehydration and may prevent metabolic acidosis (refer to Table 3).

The pancreas is the primary organ involved in the production of insulin, which is at the center of glucose homeostasis. Insulin is made in the beta cells of the pancreas. Thus, the histology of the pancreas is very important in diabetic studies. Autoimmune destruction of pancreatic cells has been reported as a component of the pathogenesis of diabetes mellitus, which is characterized by interlobular duct occlusion [42] among other histological manifestations, as seen in the diabetic control. Administration of MH-LLV showed a pancreas with intralobular ducts (IDs) and exocrine secretory acini, which characterize an intact pancreas [43] and suggest the ability of the MH-LLVs to restore the integrity of pancreatic cells towards normal on continued treatment (refer to Figure 5).

## 4. Materials and Methods

### 4.1. Schematic Overview of Experimental Program

**Figure 6:**
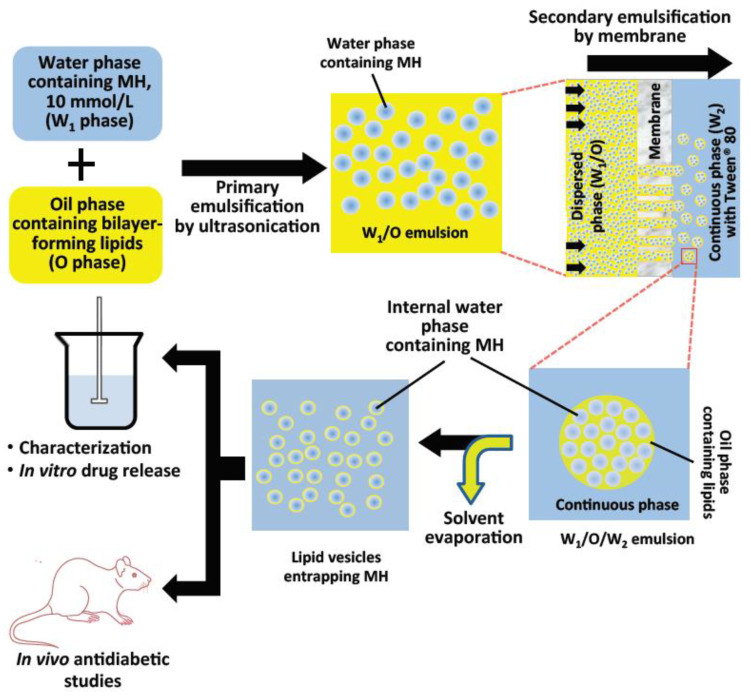
Schematic presentation of the method for the formulation of lipid vesicles encapsulating metformin hydrochloride and the in vitro and in vivo studies.

The primary emulsification step by ultrasonication produced the water-in-oil emulsion. This was followed by secondary membrane emulsification producing the water-in-oil-in-water multiple emulsion. Formation of the lipid vesicles was achieved by evaporation of oil in the organic phase of the multiple emulsions. Average diameter, the entrapment yield, and in vitro drug release were determined using standard methods. Effects of MH-LLV on serum glucose level, biochemical parameters, and pancreatic histology were evaluated by standard methods. The schematic of the preparatory process is shown in Figure 6.

### 4.2. Chemicals and Reagents

Metformin hydrochloride was purchased from LKT Laboratories, Inc (St. Paul, MN). Phospholipon^®^ 90G (P90G) was obtained from Phospholipid GmbH (Köln, Germany); oleic acid (OA) was purchased from Kermel (Mumbai, India); Tween^®^ 80 and cholesterol (Chol) were sourced from Wako Pure Chemical Industries (Osaka, Japan); sodium dihydrogen phosphate dihydrate (NaH_2_PO_4_.2H_2_O) and disodium hydrogen phosphate dodecahydrate (Na_2_HPO_4_.12H_2_O) were products of Guangdong Guanghua Science Technology Co. Ltd. (Shantou, China); and alloxan (Hydrate)-A110109 was purchased from Qualikems (Delhi, India). Picric acid was obtained from Merck (Darmstadt, Germany). N-hexane was procured from LOBA Chemie PVT Ltd. (Mumbai, India). Distilled water was supplied by the Department of Pure and Industrial Chemistry, University of Nigeria, Nsukka.

### 4.3. Methods

#### 4.3.1. Preparation of Metformin Hydrochloride-Loaded Lipid Vesicles

Lipid vesicle formulation entrapping metformin hydrochloride was performed using the multiple emulsion and solvent evaporation integrated process as described below.

Step 1: Formulation of primary (water-in-oil, W_1_/O) emulsion.

The primary emulsion, W_1_/O, was prepared as follows: first, the water (dispersed) phase (W_1_) was prepared by dispersing MH solution (W_1_, 0.010 mol/L in phosphate buffer 0.050 mol/L, pH 7.4) into a continuous oil phase (O, comprising n-hexane solution of P90G, Chol, and OA—0.026 mol/L each) at a ratio of 1:3 in a glass vial. The mixture was homogenized by sonication for 10 min with a probe-type sonicator (Athena Technology, Mumbai, India) at below 25 °C.

Step 2: Preparation of W_1_/O/W_2_ multiple emulsions by emulsification using membrane.

The membrane emulsification (ME) technique was employed in the preparation of W_1_/O/W_2_ multiple emulsion droplets. The W_1_/O phase was forced through a 1.0-mm pore-sized hydrophilic polytetrafluoroethylene (PTFE) Omnipore membrane filter sourced from Merck Millipore (County Cork, Ireland) fitted in the KS 13 Advantec filter holder by Toyo Roshi Kaisha Ltd. (Tokyo, Japan) and dispersed into the continuous phase, W_2_ consisting of aqueous phosphate buffer (0.050 mol/L, pH 7.4) solution of Tween^®^ 80 (0.1 wt %) as the emulsifier at a ratio of 1:9 (*v/v*). The W_1_/O/W_2_ formed was collected in a glass bottle.

Step 3: Solvent evaporation—formation of lipid vesicles.

Lipid vesicle suspension was formed from the multiple emulsion droplets by removal of the organic solvent under ambient condition for 20 h.

#### 4.3.2. Characterization of the Lipid Vesicles

The average sizes of the primary emulsification product (W_1_/O) and also the lipid vesicles formed were determined using a dynamic light scattering machine (DLS, Zetasizer Nano ZS, Malvern Instruments, Worcester, UK). The intensity versus size distributions of the particles were presented. The entrapment yield (EY) of metformin hydrochloride in the prepared lipid vesicles was determined using UV–Vis spectrophotometric detection of the drug. Equal volumes of methanol and lipid vesicle suspension were mixed together in a vial, a treatment that leads to the destruction of the vesicle membranes, releasing their contents into the medium. The solution was then subjected to centrifugal filtration for 25 min at 4000 rpm using a Centrisart tube (Sartorius, Germany) with 10 kDa molecular weight cut-off. Analysis of the clear permeate obtained was performed using UV–Vis spectrophotometer (Jenway, model 6504, UK) at 236 nm to give total concentration (C*_total_*) of the free drug and the entrapped ones. For the concentration of the free/unentrapped drug (C*_out_*), a mixture of the vesicle suspension and phosphate buffer was made and subjected to the above filtration condition. The lipid vesicles were precipitated and the permeate analyzed at 236 nm using a UV–Vis spectrophotometer.

The entrapment yield, EY, was therefore calculated using the formula:

(1) $$\mathrm{Entrapment}\;\mathrm{yield}\;(\%)\;=\;\frac{C_{total}-C_{out}}{C_{total}}\times 100$$

#### 4.3.3. Metformin Hydrochloride In Vitro Drug Release Study

A dialysis membrane was employed for evaluation of the in vitro release profile of metformin hydrochloride entrapped in the lipid vesicle (MH-LLV). Lipid vesicle suspension in a dialysis membrane (MWCO 12 KDa, Himedia, India) was kept in a beaker of phosphate-buffered saline (PBS, 200 mL, pH 7.4) at 37 ± 1 °C and stirred at 250 rpm with a magnetic stirrer. At 1, 2, 4, 6, and 12 h, samples (5 mL) were removed and replaced with the same amount of PBS for sink conditions to be maintained during the study period. Analysis of samples for metformin hydrochloride content was performed via UV–Visible spectrophotometric measurements (Jenway, model 6405, Essex, UK) at 236 nm.

#### 4.3.4. In Vivo Antidiabetic Studies

##### Animals, Feeding, and Ethical Guidelines

Twenty-five (25) healthy adult albino male Wistar rats of weights ranging from 150 to 200 g were supplied by the Zoological Garden of the Department of Zoology and Environmental Biology, University of Nigeria, Nsukka. The rats were transferred to and acclimatized for one week in the Department of Biochemistry’s Animal House, and this was performed under ambient environmental conditions, maintaining a 12-h light/dark cycle prior to the investigation. Essentially, the animals were fed standard animal feed (Supreme finisher feeds) and clean water inside cages with proper ventilation. The animals were handled in accordance with the ethical guidelines on the use of laboratory animals established by the Faculty of Biological Sciences’ Ethics and Biosafety Committee, University of Nigeria, Nsukka, with reference UNN/FBS/EC/1047, and in compliance with the International Standard for the use of laboratory animals [44].

##### Induction of Diabetes

The animal experiments were implemented according to the National Institute of Health (NIH) guide for care and use of laboratory animals. After 12 h (overnight) fasting of the experimental animals with free access to water, their blood glucose was checked before inducing diabetes. Single intraperitoneal injection of the experimental animals with alloxan monohydrate (150 mg/kg body weight) in normal saline was used to induce diabetes in the rats. The rats were fed normally after inducing diabetes. Seventy-two (72) hours post-induction of diabetes, the blood of the rats was checked to determine if they were diabetic. Body weight changes were monitored for 7 days after administration in addition to other symptoms of diabetes. A blood glucose concentration of 200 mg/dL and higher was labeled diabetic and the animals were selected and grouped randomly into five groups, each with five animals, as follows: NCt: Normal control; DBCt: Diabetic control; DMH: Diabetic + 5 mg/kg body weight free MH (standard control); DMLL: Diabetic + low dose (2.5 mg/kg body weight MH-LLV); DMLH: Diabetic + high dose (5 mg/kg body weight MH-LLV). The MH-LLVs and the standard free drug were administered to the rats orally once daily for seven days.

Determination of the blood glucose level was carried out before induction of diabetes (baseline glucose level), three days after induction of diabetes (day 1), and one week post-diabetes treatment (day 8) using a glucometer (Accu-Chek Active) and glucometer Strips (Trinder, 1969). The rat’s tail was slit with a surgical blade and a drop of blood was used to determine the blood glucose level. The animals were starved for 12 h prior to glucose level determination. After one week of treatment, samples of animal blood were drawn from the retro bulbar plexus of the median canthus of the eye into plain sample tubes. The blood samples were maintained at ambient condition for 30 min to achieve clotting. Centrifugation was achieved at 3000 rpm for 10 min. The clear supernatant was then carefully separated and used for the determination of lipid profile, liver marker enzymes, kidney profile, oxidative stress status, serum electrolytes, and pancreatic tissue histopathological examination of experimental animals using standard methods.

### 4.4. Statistical Analysis

IBM Statistical Product and Service Solution (SPSS) version 20.0 for Windows was used for the data analysis. Specifically, either a one-way or two-way analysis of variance (ANOVA) was applied to establish significant differences between the observed values and normal values. Resultant data were expressed as mean ± standard deviation (SD). The level of statistical significance was accepted at *p* < 0.05 (95% confidence interval).

## 5. Conclusions

A potential enhancement targeting the therapeutic efficacy of the biguanide metformin hydrochloride loaded in lipid vesicles (MH-LLVs) on alloxan-induced diabetic rats has been investigated. A sustained release of metformin hydrochloride entrapped in lipid vesicles formed through the multiple emulsification and solvent evaporation integrated process could effectively reduce hyperglycemia in alloxan-induced diabetic animals. Through this, the biochemical biomarkers investigated could be restored to near normal. This could be a result of possible improvement in the bioavailability of metformin entrapped in lipid vesicles through sustained release of the drug. Overall, the MH-LLV herein appears to be an encouraging extended-release formulation, with enhanced bioavailability, sustained release, as well as improved antihyperglycemic potentials. The direction of future studies should be to elucidate the mechanism(s) at the molecular level of the results obtained in this current study.

## Figures and Tables

**Table 1:** Serum lipid profile and liver function enzymes in different groups of animals at the end of the experimental period.

Parameters/Groups	NCt	DBCt	DMH	DMLL	DMLH
Total cholesterol (mmol/L)	2.75 ± 0.64 ^a^	4.70 ± 0.13 ^c^	3.91 ± 0.35 ^b^	2.17 ± 0.17 ^a^	2.46 ± 0.46 ^a^
Triacylglycerols (mmol/L)	1.43 ± 0.29 ^a^	2.47 ± 0.03 ^b^	1.69 ± 0.03 ^a^	1.64 ± 0.64 ^a^	1.58 ± 0.58 ^a^
HDL-chol. (mmol/L)	1.15 ± 0.54 ^b^	0.75 ± 0.05 ^a^	1.10 ± 0.03 ^ab^	1.06 ± 0.06 ^ab^	1.18 ± 0.18 ^ab^
LDL-chol. (mmol/L)	1.26 ± 0.69 ^a^	2.89 ± 0.78 ^b^	1.72 ± 0.73 ^a^	0.76 ± 0.05 ^a^	0.96 ± 0.06 ^a^
AST (U/min/mL)	13.11 ± 1.07 ^a^	19.89 ± 0.84 ^d^	16.66 ± 0.66 ^c^	15.33 ± 0.33 ^b^	13.54 ± 0.46 ^a^
ALT (U/min/mL)	10.21 ± 0.51 ^a^	16.67 ± 0.01 ^d^	15.29 ± 0.61 ^cd^	14.66 ± 0.66 ^c^	12.17 ± 1.49 ^b^
ALP (U/min/mL)	14.90 ± 0.46 ^a^	19.28 ± 0.22 ^d^	15.53 ± 0.52 ^ab^	18.00 ± 1.00 ^c^	16.65 ± 0.65 ^b^
Total bilirubin (mg/dL)	0.51 ± 0.03 ^b^	0.65 ± 0.04 ^c^	0.48 ± 0.03 ^b^	0.39 ± 0.01 ^a^	0.45 ± 0.05 ^b^

Data of measurements from five animals are presented as mean ± standard deviation (SD). ^a–d^ Presence of the same superscripts in a column indicates no significant difference (*p* > 0.05), while different superscripts in a column indicate significant difference (*p* < 0.05). *NCt: Normal control; DBCt: Diabetic control; DMH: Diabetic + 5 mg/kg body weight free MH; DMLL: Diabetic + low dose (2.5 mg/kg body weight MH-LLV); DMLH: Diabetic + high dose (5 mg/kg body weight MH-LLV); HDL-chol.—high-density lipoprotein cholesterol; LDL-chol.—low-density lipoprotein cholesterol; AST – aspartate aminotransferase; ALT – alanine aminotransferase, ALP – alkaline phosphatase.

**Table 2:** Oxidative stress status of the animals in different groups at the end of the experimental period.

Parameters/Groups	NCt	DBCt	DMH	DMLL	DMLH
GSH (mg/dL)	3.10 ± 0.19 ^a^	2.46 ± 0.44 ^a^	3.51 ± 0.10 ^a^	2.48 ± 1.49 ^a^	4.03 ± 0.08 ^b^
SOD (U/min/mL)	1.12 ± 0.02 ^b^	1.02 ± 0.08 ^ab^	1.12 ± 0.01 ^b^	0.91 ± 0.08 ^a^	1.12 ± 0.09 ^b^
CAT (U/min/mL)	1.77 ± 0.05 ^b^	1.07 ± 0.10 ^a^	1.48 ± 0.04 ^ab^	1.39 ± 0.46 ^ab^	1.49 ± 0.18 ^ab^
GPx (U/min/mL)	6.38 ± 0.44 ^c^	4.64 ± 0.35 ^a^	5.50 ± 0.44 ^abc^	5.04 ± 0.88 ^ab^	6.03 ± 0.86 ^bc^
MDA (mg/dL)	2.49 ± 0.28 ^b^	3.32 ± 0.19 ^c^	3.16 ± 0.03 ^c^	2.21 ± 0.37 ^ab^	2.01 ± 0.19 ^a^

Data of measurements from five animals are presented as mean ± standard deviation (SD). ^a–c^ Presence of similar superscripts in a column indicates no significant difference (*p* > 0.05), while different superscripts in a column indicate significant difference (*p* < 0.05). Please refer to the footnotes of Table 1 for the description of abbreviations. GSH—glutathione; SOD – superoxide dismutase; CAT – catalase; GPx – glutathione peroxidase; MDA – malondialdehyde.

**Table 3:** Renal function status and serum electrolyte levels in different groups of animals at the end of the experimental period.

Parameters/Groups	NCt	DBCt	DMH	DMLL	DMLH
Creatinine (mg/dL)	0.92 ± 0.08 ^ab^	0.68 ± 0.11 ^a^	0.88 ± 0.01 ^ab^	1.00 ± 0.10 ^b^	1.07 ± 0.07 ^c^
Urea (mg/dL)	21.03 ±1.12 ^ab^	26.22 ± 3.40 ^b^	24.22 ± 0.52 ^ab^	24.29 ± 0.29 ^ab^	19.40 ± 5.39 ^a^
Sodium (mmol/L)	113.67 ± 0.88 ^b^	100.33 ± 7.97 ^a^	115.00 ± 0.58 ^b^	120.00 ± 2.89 ^b^	119.00 ± 0.58 ^b^
Potassium (mmol/L)	3.28 ± 0.40 ^b^	2.83 ± 0.09 ^a^	3.28 ± 0.02 ^b^	3.28 ± 0.16 ^b^	3.33 ± 0.19 ^b^
Chloride (mmol/L)	78.90 ± 1.23 ^b^	64.10 ± 9.17 ^a^	81.15 ± 0.11 ^b^	79.44 ± 0.28 ^b^	81.74 ± 0.43 ^b^
Bicarbonate (mmol/L)	24.05 ± 0.23 ^b^	19.83 ± 1.95 ^a^	24.31 ± 0.20 ^b^	23.03 ± 0.17 ^b^	24.55 ± 0.32 ^b^

Data of measurements from five animals are presented as mean ± standard deviation (SD). ^a–c^ Presence of the same superscripts in a column indicates no significant difference (*p* > 0.05), while different superscripts in a column indicate significant difference (*p* < 0.05). Please refer to the footnotes of Table 1 for the description of abbreviations.

## Data Availability

Data sharing is not applicable.
